# Human extrahepatic and intrahepatic cholangiocyte organoids show region-specific differentiation potential and model cystic fibrosis-related bile duct disease

**DOI:** 10.1038/s41598-020-79082-8

**Published:** 2020-12-14

**Authors:** Monique M. A. Verstegen, Floris J. M. Roos, Ksenia Burka, Helmuth Gehart, Myrthe Jager, Maaike de Wolf, Marcel J. C. Bijvelds, Hugo R. de Jonge, Arif I. Ardisasmita, Nick A. van Huizen, Henk P. Roest, Jeroen de Jonge, Michael Koch, Francesco Pampaloni, Sabine A. Fuchs, Imre F. Schene, Theo M. Luider, Hubert P. J. van der Doef, Frank A. J. A. Bodewes, Ruben H. J. de Kleine, Bart Spee, Gert-Jan Kremers, Hans Clevers, Jan N. M. IJzermans, Edwin Cuppen, Luc J. W. van der Laan

**Affiliations:** 1 Department of Surgery, Erasmus MC-University Medical Center, Wytemaweg 80, 3015 CN Rotterdam, The Netherlands; 2 Hubrecht Institute for Developmental Biology and Stem Cell Research, KNAW and University Medical Center Utrecht, Utrecht, The Netherlands; 3 Center for Molecular Medicine and Oncode Institute, University Medical Center Utrecht, Utrecht, The Netherlands; 4 Department of Gastroenterology, Erasmus MC-University Medical Center, Rotterdam, The Netherlands; 5 Goethe-University Frankfurt, Buchmann Institute for Molecular Life Sciences, Frankfurt, Germany; 6 Department of Metabolic Diseases, Wilhelmina Children’s Hospital, University Medical Centre Utrecht, Utrecht, The Netherlands; 7 Department of Neurology, Erasmus MC-University Medical Center, Rotterdam, The Netherlands; 8 Department of Pediatric Gastroenterology Hepatology and Nutrition, University Medical Center Groningen, University of Groningen, Utrecht, The Netherlands; 9 Department of Hepato-Pancreato-Biliary Surgery and Liver Transplantation, University Medical Center Groningen, University of Groningen, Groningen, The Netherlands; 10 Department of Clinical Sciences of Companion Animals, Faculty of Veterinary Medicine, Utrecht University Utrecht, Utrecht, The Netherlands; 11 Erasmus Optical Imaging Centre, Erasmus MC-University Medical Center, Rotterdam, The Netherlands

**Keywords:** Cell biology, Molecular biology, Stem cells, Gastroenterology

## Abstract

The development, homeostasis, and repair of intrahepatic and extrahepatic bile ducts are thought to involve distinct mechanisms including proliferation and maturation of cholangiocyte and progenitor cells. This study aimed to characterize human extrahepatic cholangiocyte organoids (ECO) using canonical Wnt-stimulated culture medium previously developed for intrahepatic cholangiocyte organoids (ICO). Paired ECO and ICO were derived from common bile duct and liver tissue, respectively. Characterization showed both organoid types were highly similar, though some differences in size and gene expression were observed. Both ECO and ICO have cholangiocyte fate differentiation capacity. However, unlike ICO, ECO lack the potential for differentiation towards a hepatocyte-like fate. Importantly, ECO derived from a cystic fibrosis patient showed no CFTR channel activity but normal chloride channel and MDR1 transporter activity. In conclusion, this study shows that ECO and ICO have distinct lineage fate and that ECO provide a competent model to study extrahepatic bile duct diseases like cystic fibrosis.

## Introduction

Integrity of the biliary tree is imperative for liver function. Diseases that affect the bile duct integrity are common and often life-threatening. Although the adult liver is well-known for its regenerative capacity, the cellular events that drive repair of the bile duct system is not fully elucidated. Mature cholangiocytes are known to have self-renewal capacity, both to maintain homeostasis and in response to bile duct injury^1–4^. In larger bile ducts, including in the extrahepatic bile ducts (eBD), peribiliary glands are thought to harbor biliary stem/progenitor cells. Evidence suggests that these peribiliary glands provide a proliferative response upon damage of the bile duct providing new cholangiocytes to restore the biliary lining^5^. These biliary progenitor cells have a different embryological origin than the bipotent progenitors found in the liver and share a common developmental origin with the pancreas and duodenum^6,7^. This distinct developmental lineage might explain the differences in morphology, function and proliferation between intra- and extrahepatic cholangiocytes^8–11^.

With the development of the 3D organoid culture technique^12^, epithelial cells, including those found in the liver^13,14^ can be expanded in vitro driven by canonical Wnt-signaling. The original liver organoids were shown to be derived from LGR5 and EpCAM double positive cells located in the intrahepatic bile ducts (iBD), both for human and mouse. More recently, work from the human liver cell atlas further specified the liver organoid-initiating cells as the TROP2 intermediate-EpCAM positive subset of cholangiocytes which share characteristics with biliary stem/progenitor cells^15^. Therefore, the original liver organoids^14^ are derived from iBD cells and should correctively be termed IntrahepaticCholangiocyte Organoids (ICO). However, these ICO organoids might not recapitulate unique features of eBD cells and might therefore not be the favorable source to be used to model extrahepatic biliary diseases like cholangiocyte dysfunctions, biliary strictures and atresia.

More recently, a novel culture method was published by Sampaziotis and colleagues^16^, to expand primary cholangiocyte-like cells as organoids by stimulation of the non-canonical Wnt signaling of the planar cell polarity pathway. These organoids retained many biliary characteristics in culture and apart from their application in disease modeling, they were used to bioengineer artificial ducts that could be successfully transplanted in mice. However, these non-canonical Wnt stimulated cholangiocyte organoids, unlike conventional ICO, do not express stem cell markers (including LGR5), but do express markers of mature cholangiocytes. In parallel with our studies, Rimland et al. recently showed that cholangiocyte organoids isolated from intrahepatic ducts were different when compared to cholangiocyte organoids derived from common bile duct, gallbladder, and pancreatic duct. Strikingly, they found that only a limited number of regional-specific gene markers of the tissue were conserved in the organoids, possibly driven by the culture conditions they were subjected to Ref.^17^. Only expression of common bile duct specific gene, HOXB2, was retained in organoids derived from the common bile duct and not in those derived from pancreas duct or gall bladder.

The aim of this study was to establish Wnt-dependent organoids from human eBD and compare these to paired ICO from liver samples of the same donor. Here we report that Wnt-stimulated Extrahepatic Cholangiocyte Organoids (ECO) have distinct differentiation capacity towards hepatocyte-fate when compared to ICO. As proof of qualifying as model for eBD disease, we showcase ECO from a cystic fibrosis (CF) patient.

## Results

### Efficient organoid initiation from the human common bile duct

**Figure 1:**
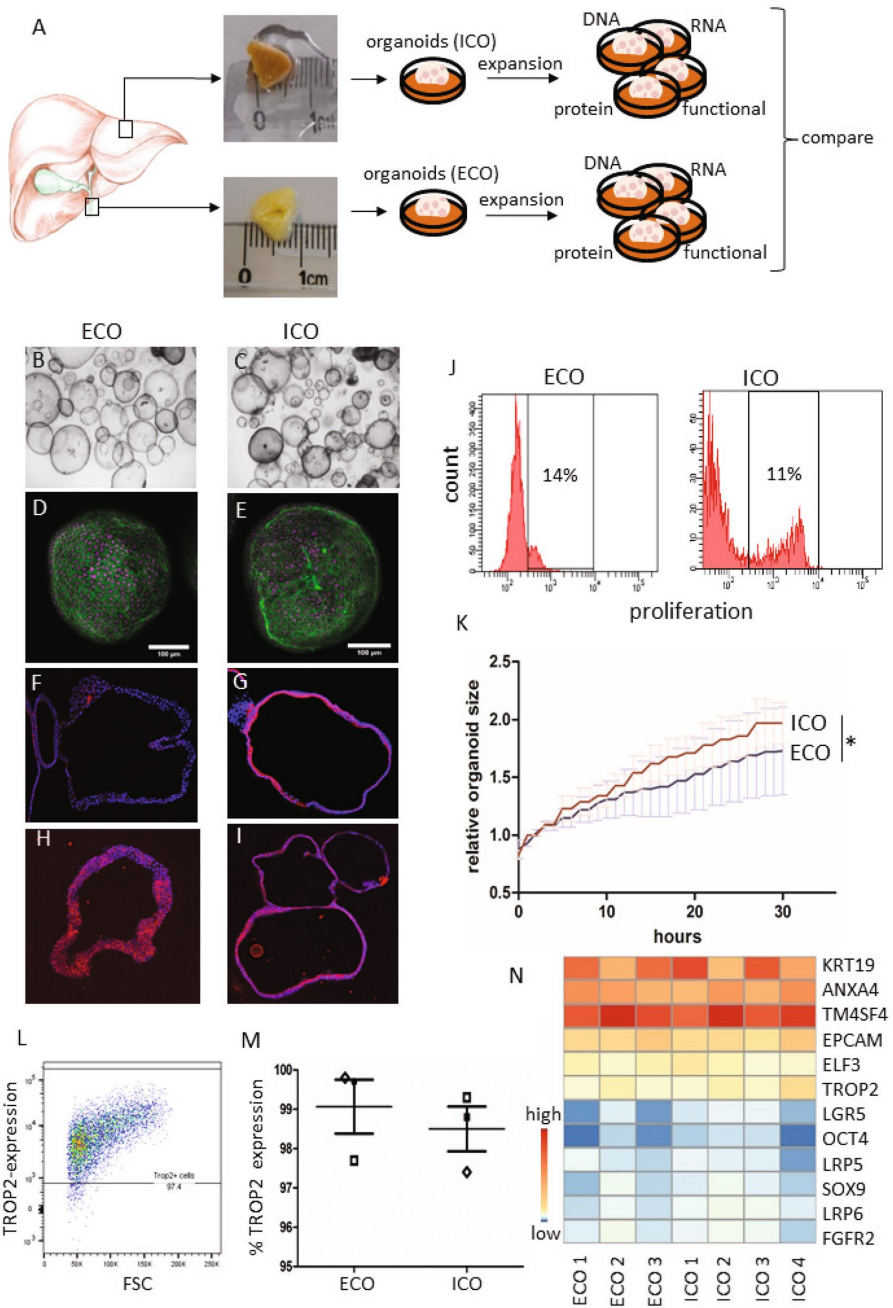
Culture initiation and characteristics of liver- and extrahepatic bile duct-derived organoids. Schematic representation of organoid culture initiation from liver biopsies (intrahepatic bile duct; iDO) and extrahepatic bile duct (eDO) (**A**). Organoids were cultured and passaged weekly (1:3 for over 30 passages/ > 8 mo) and harvested at different passages for further analysis. ECO were cultured as efficiently as liver-derived ICO. Both look similar at the microscopic level (B, C), when stained with phalloidin/DAPI (**D**,**E**), and in FFPE sections stained by immunofluorescent KRT7 (**F**,**G**—10×) and KRT19 (**H**,**I**—10×). EdU-incorporation showed a similar percentage of proliferating cells (11–14% in S-phase) in both organoid types at passage 10. Shown is one representative result of three paired donor lines tested (**J**). The individual percentages are shown in Supplemental Fig. S2. Despite no significant difference in proliferation (*p* = 0.122), a significant difference in organoid size was found when analyzing individual organoids for 30 h (**K**) Shown is mean ± SEM of 100 organoids from paired ECO and ICO of three different donors. TROP2 protein expression was found in almost 100% of the organoid cells, both for ECO and ICO (**L**,**M**) (three paired organoid lines). Expression heatmap of selected genes described by Aizarani et al., as markers for liver progenitors^15^. The expression levels per gene were collected from the normalized RNA sequencing analysis performed on four ICO and three ECO lines, and visualized as Z-score. Though the level of expression varied per gene, no significant difference in expression was found between ICO and ECO (**N**).

Samples from donor liver and the common bile duct (eBD) were dissociated and used for culture initiation (Fig. 1A). EpCAM and LGR5 frequencies were determined in the cells that were isolated from eBD and liver biopsies. Flow cytometric analysis show that EpCAM levels were low 1.2 ± 1.6% in eBD and 0.9 ± 0.7% (average ± SD) in liver starting populations as anticipated and as previously shown^18^. Cells isolated from ICO and ECO expressed ~ 100% EpCAM as shown before^14^ (Supplementary Fig. S1). LGR5 expression was too low to be detected by flow cytometry (not shown). Organoids could be initiated from human eBD tissue with > 95% success rate from over 40 biopsies from both male and female organ donors or patients with a wide age range (16–70 years). On average, the first ECO were visible in cultures at 7 days (6.8 days ± 1.0 SD). Organoids were grown 3-dimentional in hydrogels (Matrigel) and after initiation organoid cultures were passaged one to two times per week (split 1:3). Organoid initiation efficiency and expansion potential was similar to paired ICO cultured from liver biopsies from the same donor (data not shown). In accordance with previous results^14^, ECO could be viably frozen and thawed and in general, behaved similarly to ICO. Several organoid lines were biobanked early but many ECO cultures (n = 15) were passaged over 30 times for a period of 8 months or longer without clear signs of exhaustion. The ICO and ECO cultures were morphologically similar and no apparent histological differences were found (Fig. 1B–I). Both cultures remained proliferative at late passages (> 6 months), with 11.0 ± 2.1% and 17.3 ± 2.4% (mean ± SEM) of the cells in S-phase at P10, respectively. Additional Ki67 stainings of ECO and ICO organoid sections confirmed this (Fig. 1J and Supplementary Fig. S2), and were similar to previously described percentages for ICO^14^. Despite similar proliferation rates, the increase of relative organoids size as measured by time-lapse bright-field microscopy was significant differences for paired organoid types (Fig. 1K). Organoid size was measured starting 3.5 days after splitting and followed for 30 h. After 12 h of measuring the size of ICO were significantly larger than ECO from the same donor (n = 100 organoids per type, *p* < 0.001). As shown in Fig. 1L, both organoid types express the trophoblast cell surface protein 2 (TROP2) protein which is associated with organoid-initiating cells) and no significant difference was found in TROP2 protein expression (Fig. 1M, 99.1% ± 1.2% in ECO and 98.5% ± 1.0% in ICO; *p* = 0.67). Together, these results show that organoids are initiated efficiently from human eBD and iBD and have similar characteristics with only a small but significant differences in organoid size expansion.

### Gene expression analyses of ECO and ICO

When looking at the expression of specific genes known to be markers for intrahepatic organoid-initiation cells identified by single cell RNA sequencing and cell sorting^15^, all genes were present and no significant difference in expression was observed between ECO and ICO (Fig. 1N). These genes include epithelial cell adhesion molecule (EpCAM), leucine-rich repeat-containing G-protein coupled receptor 5 (LGR5), the low-density lipoprotein receptor-related protein (LRP5/6) co-receptor group^12^, tumor-associated calcium signal inducer 2 (TACSTD2 or TROP2), SRY-Box 9 (SOX9), Cytokeratin 19 (KRT19), fibroblast growth factor receptor 2 (FGFR2), transmembrane 4 L Six Family member 4 (TM4SF4) and others^15^. Though all these markers were found positive in organoids, some genes were highly expressed (TM4SF4, KRT19) and some genes relatively low (LGR5 and OCT4).

**Figure 2:**
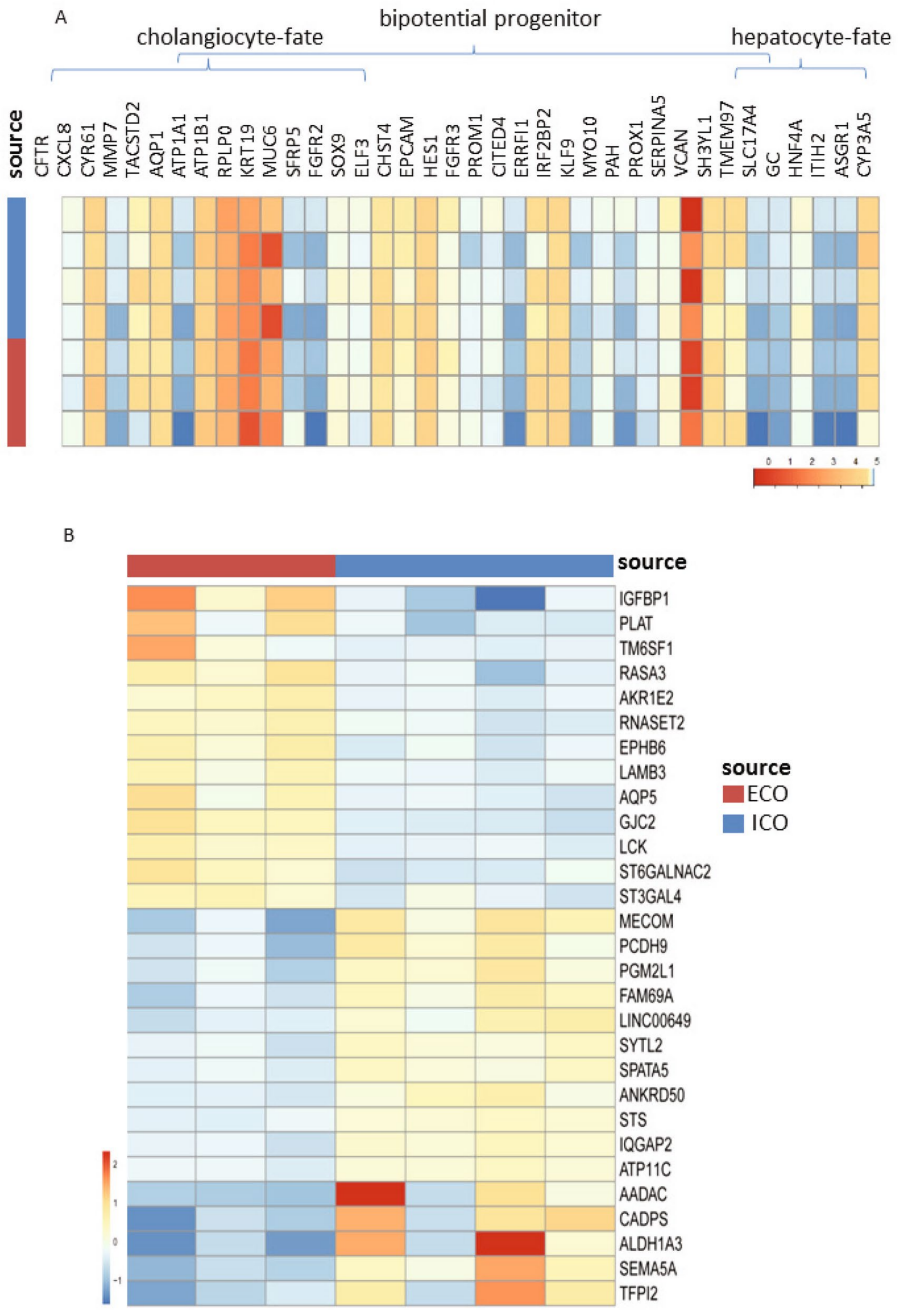
Limited differences in gene expression between ECO and ICO. Gene expression analysis of three paired ICO and ECO and one additional ICO line was performed using RNAseq. Selected genes, as described by Aizarani et al.^15^ that are expressed in EpCAM-positive cells and associated with progenitors, hepatocyte-fate or cholangiocyte-fate. Shown are normalized (bulk) RNA sequencing results of the undifferentiated ECO and ICO for this specific gene set. Expression of the progenitor, hepatocyte-fate- and cholangiocyte-fate associated genes was similar between ECO and ICO. The expression heatmap shows Z-scores that are color-coded as shown (**A**). No significant differences between ICO and ECO for this gene set was observed. The expression heatmap (**B**) indicates clusters of genes that are differentially expressed (29 in total, *p* adjusted < 0.05), 13 higher expressed in ECO and 16 higher in ICO. This analysis reveals that although both organoid types are very similar in gene expression profiles, there are some differences albeit in a relatively low number of genes.

Extending the gene expression analysis of specific genes associated with bi-potential progenitors, or progenitors with hepatocyte-fate or cholangiocyte-fate, again as described in the human liver atlas study^15^, did not show any significant different expressed between ICO or ECO (Fig. 2A). Looking at the global gene expression of three paired organoid sets and one additional non-paired ICO, revealed a high degree of similarity and only small differences. As shown in Fig. 2B, this RNA sequencing analyses showed that only 29 genes were significantly differentially expressed (*p* adjusted < 0.05), of which 13 were expressed at higher levels in ECO and 16 were expressed at higher levels in ICO. Among the genes that were more abundantly expressed in ECO were Aquaporin5 (AQP5), a classic water transporter-channel known to be expressed on cholangiocytes and pancreatic epithelium and to a lesser degree by hepatocytes, and Insulin Like Growth Factor Binding Protein 1 (IGFBP1) known to be involved in ductular reactions of the biliary epithelium in response to liver damage^19^. Genes associated with the alcohol dehydrogenase pathway (ALDH1A3, AADAC) and lipid metabolism (STS), typically expressed in hepatocytes, are differentially expressed and upregulated in ICO. In particular ALDH1A3, highly expressed in ICO, is known to be involved in the biosynthesis of retinoic acid (RA) and RA signaling transduction. Interestingly, a previous study with lung organoids showed that inhibition of RA pathway causes an increased ALDH expression and an increase the size of organoids^20^. Thus, higher ALDH expression in ICO may explain their larger organoid size as compared to ECO (Fig. 1K), however, this requires further experimental proof. Other hepatocyte-related genes as CYP3A4 and HNF4a were not differently expressed in ICO and ECO (Fig. 2B). The regional-specific gene for common bile duct tissue which was retained in common bile duct organoids^17^, HOXB2, was not significantly different between ECO and ICO (not shown). This same study reported that PROM1, SOX9 and Albumin were significantly higher expressed in intrahepatic versus extrahepatic bile duct organoids. These difference was not confirmed in our ICO and ECO (data not shown) and may be related to the different culture conditions. Taken together, although these results show that gene expression in ICO and ECO was generally quite similar, several genes were found to be differentially expressed.

### Cholangiocyte-related ion transport activity in ECO and ICO

**Figure 3:**
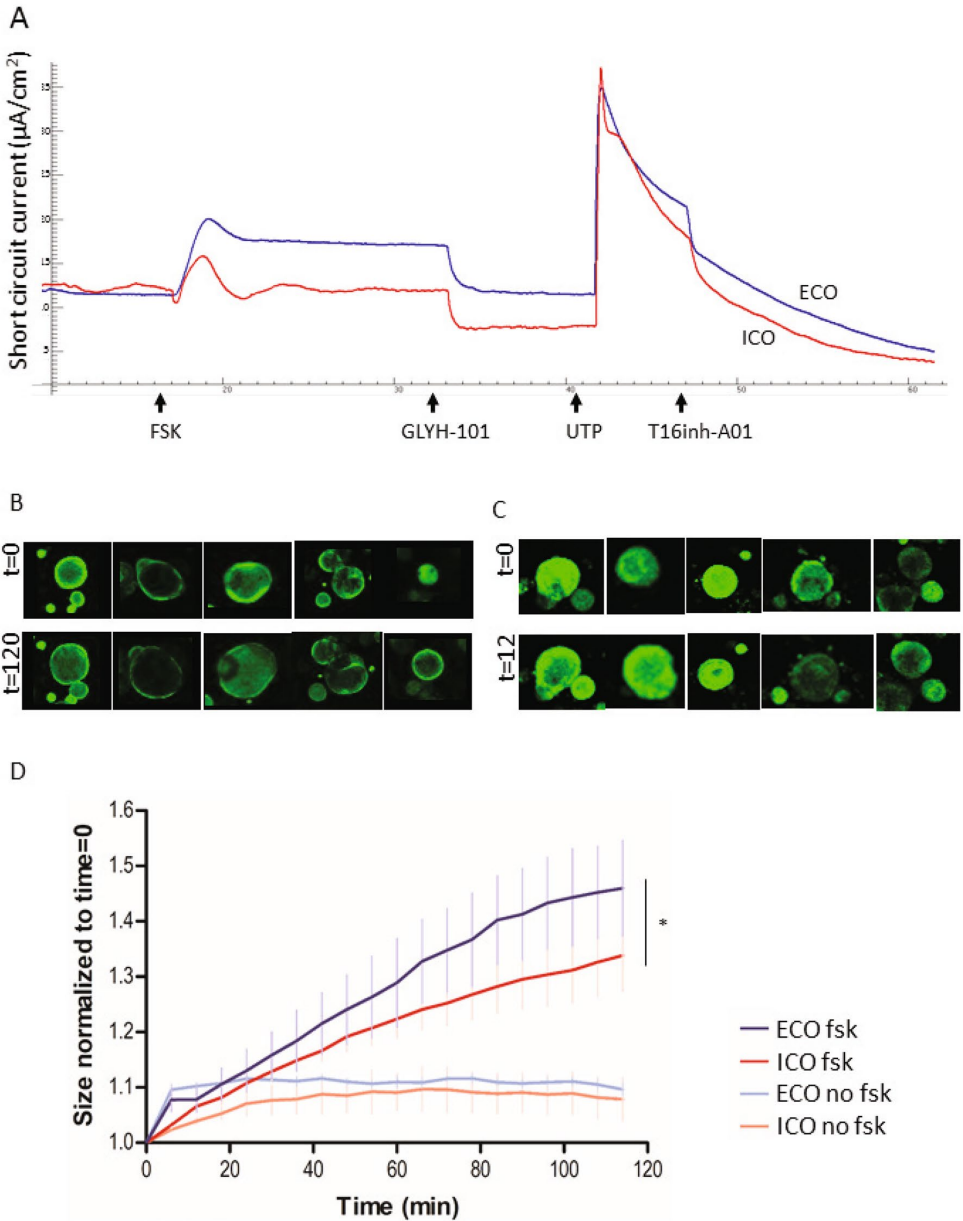
Forskolin-induced ion channel activity is more prominent in ECO then ICO. A representative Ussing experiment (**A**) shows CFTR stimulation by forskolin (FSK, 5 μM) in ECO (blue line) and ICO (red line) organoids. GlyH-101 inhibits the CFTR transporter channel specifically. Ca^2+^-activated Cl-channels (CaCC) are stimulated by UTP and inhibited by CaCC inhibitor T16inh-A01 (arrows) demonstrating the presence of functional CFTR and Ca^2+^-activated Cl-channels in these organoids. Functionality of CFTR in 3D grown organoids was shown by FIS. Representative pictures of five individual calcein green-labeled ECO (**B**) and ICO (**C**) before (t = 0) and after (t = 120) forskolin stimulation show the swelling in time. Qualification of organoid size in time showed that ECO swell significantly larger upon forskolin-activation than ICO (D) (*p < 0.0001).

To execute their function to modify hepatocyte-derived bile, cholangiocytes harbor a number of transporter-channels on their apical membranes. This include the cAMP-activated CFTR channel and the Ca^2+^-activated Cl^−^ channel CaCCs. As shown in Fig. 3, basolateral forskolin stimulation of ECO or ICO cells grown as a 2D monolayer, induced a short-circuit current (Isc). This Isc signal could be completely inhibited by the CFTR-inhibitor GlyH-101 (N-(2-naphthalenyl)-((3,5-dibromo-2,4-dihydroxyphenyl)methylene)glycine hydrazide)^21^. Furthermore, apical UTP, which elicits Ca^2+^ mobilization through activation of purinergic (P2Y) receptors, induced an lsc that was inhibited by the CaCC inhibitor T16inh-A01 (Fig. 3A). No differences were found in activation of the CaCC by UTP and subsequent inhibition by T16inh-A01. Of note, although in both organoids types clear CFTR activity was measured, the most pronounced response to forskolin was observed in ECO. This observation was further supported with another CFTR measure, the 3D forskolin-induced swelling (FIS) assay. In this FIS assay, due to changed osmolarity upon forskolin stimulation, water is transported inside the lumen, causing the organoids to swell. Figure 3B (ECO) and Fig. 3C (ICO) show the organoid size before and after 120 min forskolin stimulation. Both ECO and ICO showed swelling upon forskolin activation, demonstrating also polarization of the cells and luminal expression of the CFTR channel in the organoids. Automated quantification of the organoid-size over time confirmed significantly more swelling in ECO compared to ICO (Fig. 3D, *p* < 0.0001).

### Cholangiocyte-fate differentiation potential is similar for ECO and ICO

**Figure 4:**
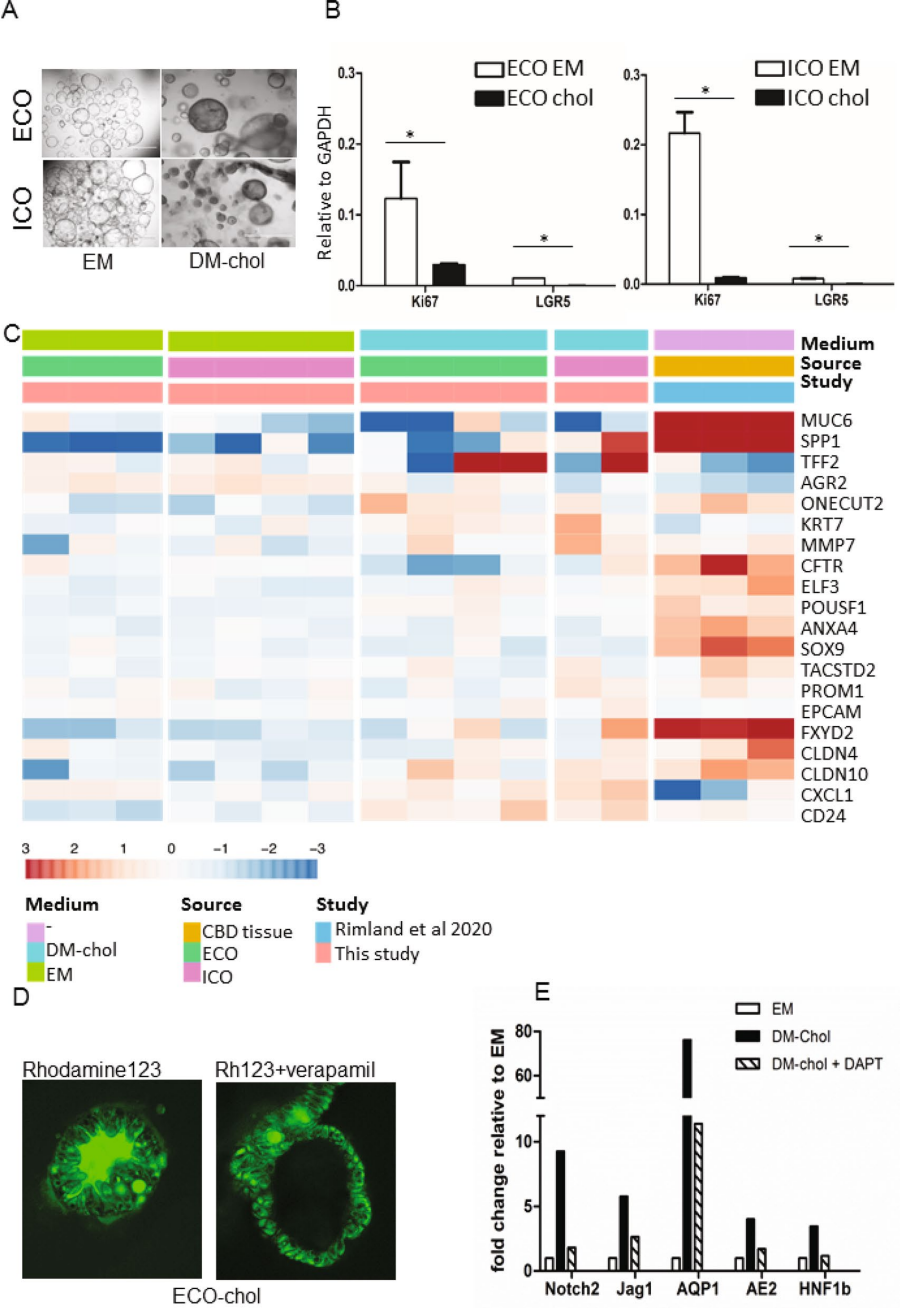
Both ECO and ICO have cholangiocyte-fate differentiation potential. Culturing organoids in cholangiocyte differentiation medium (DM-chol) for 14 days result in morphological changes, Organoids became more dense, had thicker outer walls as compared to EM condition and stopped proliferating (**A**). Shown are representative bright field microscopic images (bar = 1000 um). At gene expression level, stem cell and proliferation markers LGR5 and Ki76 were significantly downregulated in DM-chol both for ECO and ICO (**B**). Gene expression clustering is based on 20 genes selected for their high expression in cholangiocytes by single cell RNA sequencing. For benchmarking the cholangiocyte-differentiated ICO and ECO bile duct tissue gene expression profiles were used (Rimland et al.^17^). Both ICO and ECO showed clear upregulation of cholangiocyte genes in DM-chol conditions (**C**). Fluorescent tracer dye was used to determine transporter channel activity in DM-chol ECO (**D**) As shown in left panel, Rhodamine123 is actively transported into the lumen of ECO-chol. This luminal transport of rhodamine was completely inhibited by MDR1 inhibitor, verapamil (10 nM, right panel). Blocking the Notch-pathway by DAPT, inhibit cholangiocyte-fate differentiation of ECO (**E**). This confirms in vitro that Notch signals are important drives of cholangiocyte lineage development.

Different protocols have been developed for differentiation towards a cholangiocyte-like phenotype, both for iPSC and ICOs. As the starting cell population prior to differentiation already have many cholangiocyte-like properties (i.e. ion and water channel activities), this cholangiocyte-fate differentiation can also be considered maturation. As shown in Fig. 4A, after 14 days culture in differentiation medium, organoids became more dense, had thicker outer walls and stopped proliferating as shown in Fig. 4B, at gene expression level, stem cell and proliferation markers LGR5 and Ki76 were significantly downregulated in DM-chol both for ECO and ICO, confirming loss of stemness and proliferation. To benchmark the cholangiocyte-differentiated ICO and ECO, RNA expression profiles from bile duct tissue, published by Rimland et al.^17^, were used. For this, 20 genes known to be highly expressed in cholangiocytes were used. Gene expression clustering of ICO, ECO and the bile duct tissue showed that in both ICO and ECO in DM-cholangiocyte conditions, these cholangiocyte genes were upregulated (Fig. 4C).

The P-glycoprotein (Pgp) membrane transporter family member MDR1, is an apical transporter known to be expressed by cholangiocytes. Differentiated ECO readily took up the tracer dye and actively transported this to the luminal side via MDR1, resulting in a fluorescent organoid lumen (Fig. 4D). MDR1 dependency was confirmed by blocking luminal transport of Rh123 with the MDR1 antagonist Verapamil, resulting in accumulation of dye in the cell cytoplasm. Similar results were observed for ICO in DM-chol (not shown). Finally we confirmed that cholangiocyte-lineage differentiation is highly dependent on Notch signaling (Fig. 4E). For this, a specific small molecular inhibitor of the Notch pathway, DAPT, was added during the differentiation culture in DM-chol. Clearly the cholangiocyte-specific genes AQP1, AE2 and HNF1b which were upregulated during DM-chol, were completely downregulated by treatment with DAPT. Also the Notch-related genes Notch2 and Jag1 were downregulated by DAPT (Fig. 4E). These results indicate that organoids initiated from both eBD and iBD can be driven towards a similar adult cholangiocyte-like phenotype.

### Different hepatocyte-fate differentiation potential of ECO and ICO

**Figure 5:**
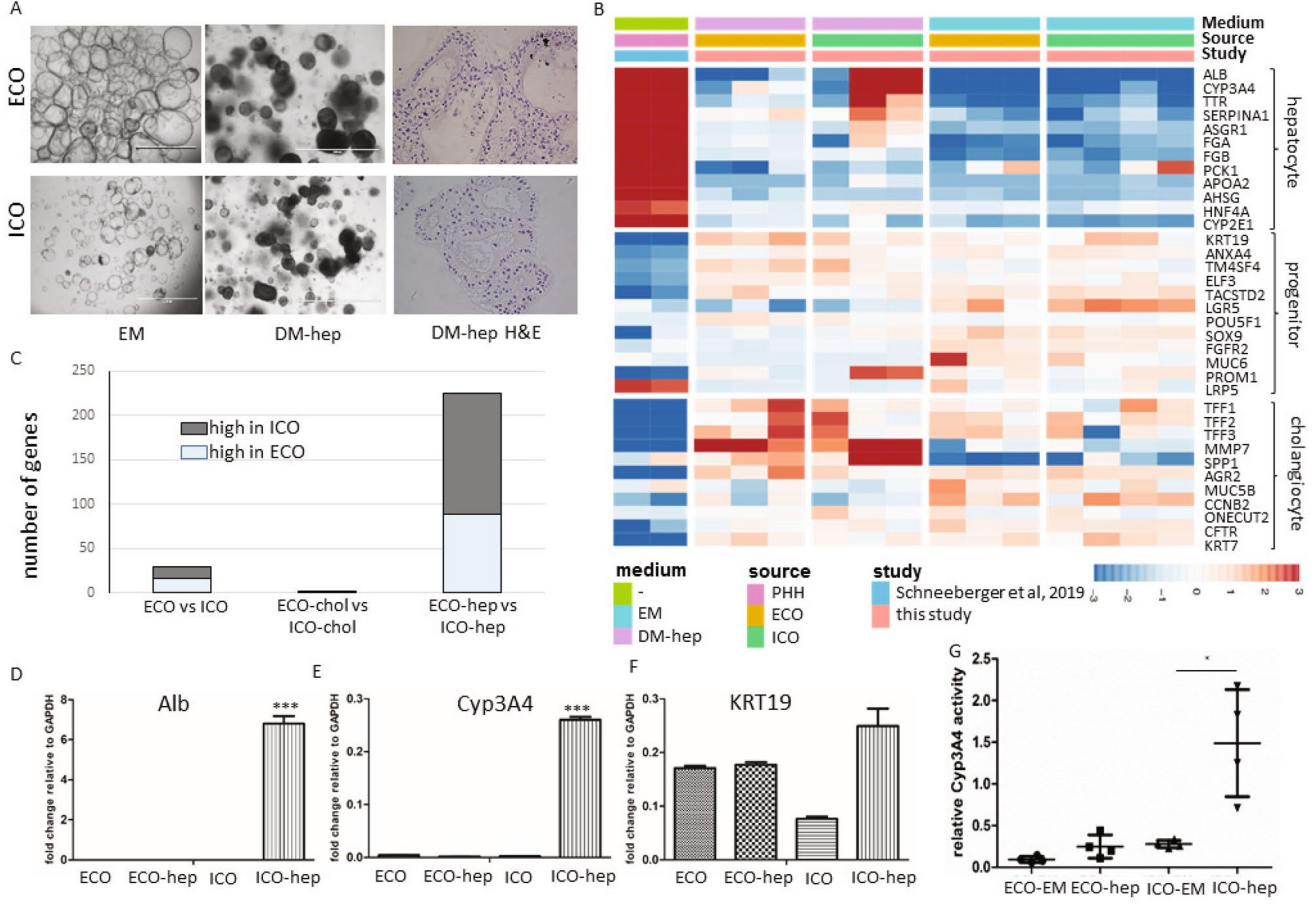
ECO lack hepatocyte-fate differentiation potential. Culturing organoids in hepatocyte differentiation medium (DM-hep) for 14 days result in morphological changes (**A**). Organoids became more dense (DM-hep) and stopped proliferating (bar = 1000 um). H&E staining of 5 µm organoid Sections (40×) show multiple cell layers and dense cellular structure in DM-hep. Bulk RNA sequencing analysis was performed and 35 selected genes associated with heptatocyte, cholangiocyte and liver progenitor cells^15^ provided an expression heatmap showing the relative expression of ECO and ICO organoids that were differentiated towards hepatocyte-fate (**B**). Relative gene expression levels are indicated in color, with blue for down-regulated genes and red for upregulated genes. None of the ECO-hep organoids clearly expressed hepatocyte-specific genes, whereas 2 of the 3 ICO-hep organoids showed expression towards a hepatocyte-like phenotype. Primary hepatocytes (PHH, adapted from Schneeberger et al.^23^), served as positive controls. Assessing general differences in ICO and ECO gene expression showed that in DM-hep condition the difference between ICO and ECO is biggest whereas small differences in gene expression was observed in DM-chol (**C**). Gene expression levels of hepatocyte-specific genes albumin (**D**) and Cyp3A4 (**E**) were further confirmed by qRT-PCR analyses in independent experiments. Both albumin (**D**) and Cyp3A4 (**E**) were again found increased in ICO and not in ECO. (**C**). KRT19 (**F**) expression levels did not differ significantly between these organoid types. Significance is indicated as ***p < 0.0005. As a functional measure of hepatocyte metabolic activity, Cyp3A4 activity was measured using a commercially available kit (**G**). The relative Cyp3A4 activity was significantly higher in DM-hep conditions for ICO but not for ECO. Significance is indicated as *p < 0.01.

Previous studies demonstrated that ICO have the potential to differentiate towards the hepatocyte lineage^14^. As shown in Fig. 5, culturing organoids in hepatocyte differentiation medium (DM-hep) results in a clear morphological change compared to organoids in expansion medium (EM) in both ICO and ECO. Histological sections of the ECO and ICO grown in DM-hep medium for 14 days, show increasing cellular density within the individual organoids (Fig. 5A). Comparative gene expression analysis of EM and DM-hep of 35 genes specific for hepatocytes, cholangiocytes or progenitor cells selected from the human liver atlas^15^ is shown in Fig. 5B. Gene expression of primary hepatocytes (PHH) as published by Schneeberger et al.^22^ were included as a benchmark for hepatocyte-fate differentiation. Expression of the hepatocyte-specific genes were clearly different between ICO and ECO. In the DM-hep condition ECO showed less upregulation of hepatocyte-specific genes as compared to ICO, whereas progenitor- and cholangiocyte-related genes were not significantly different. Of note, one of the ICO lines showed less prominent hepatocyte-fate differentiation but it is well known that differentiation potential varies between individual donors.

Assessing general differences in ICO and ECO gene expression showed that undifferentiated organoids in EM have only limited number of significantly different genes (16 genes significantly higher in ICO and 13 in ECO, shown in Fig. 2B). As shown in Fig. 5C, in the DM-chol condition the difference in gene expression between ICO and ECO completely disappeared (1 significantly different gene). However, in the DM-hep condition clearly heterogeneity in gene expression profiles increased. In ECO 136 genes were significantly higher and 89 genes were found significantly higher in ICO (Fig. 5C), indicating a difference in response to the DM-hep condition between these organoid types. The expression of hepatocyte-specific genes ALB (Fig. 5D) and CYP3A4 (Fig. 5E) was further confirmed in 3 independent differentiation experiments and quantified by qRT-PCR. Again, though progenitor-related gene KRT19 was not significantly different regulated between ICO and ECO (Fig. 5F), again only ICO showed significant upregulation of ALB and CYP3A4 in the DM-hep condition whereas ECO clearly did not upregulate ALB and CYP3A4. This was also confirmed on a functional level testing CYP3A4 activity in an enzymatic assay (Fig. 5G). Also here, only ICO and not ECO showed a significant increase in CYP3A4 activity in DM-hep condition. Together these results indicate that though both ECO and ICO have similar cholangiocyte lineage potential (Fig. 4), only ICO and not ECO show hepatocyte lineage potential. This indicate both organoid types retain some regional specific differentiation potential, with the extrahepatic bile duct region lacking clear hepatocyte-fate differentiation.

### Modelling cystic fibrosis biliary disease in ECO

**Figure 6:**
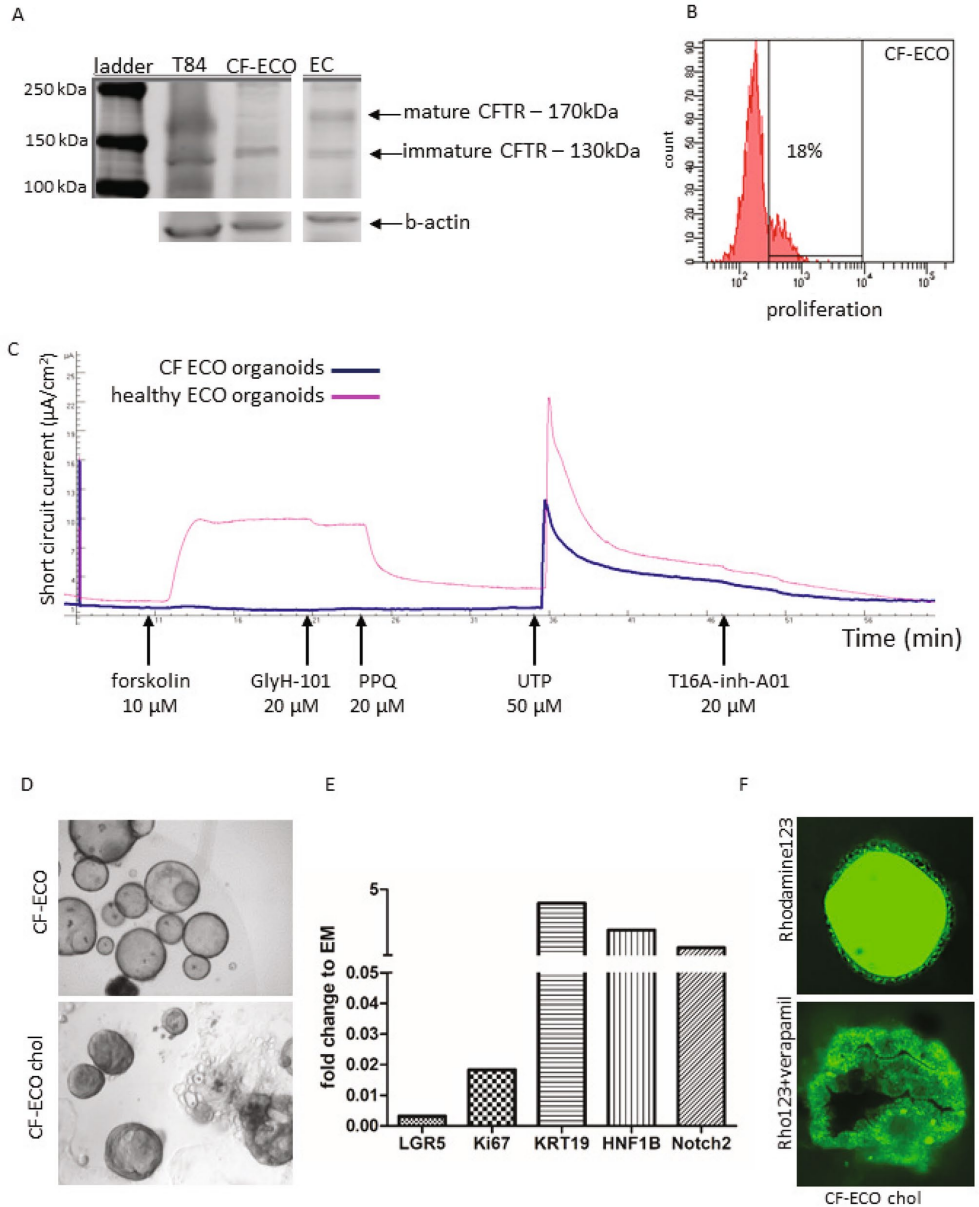
Patient-derived ECO can model cystic fibrosis biliary disease**.** ECO cultures were initiated from CF patient extrahepatic bile duct tissue. ECO from healthy donors were used as positive controls. Western blot analysis showed clear expression of wild type mature CFTR protein (170 kDa) in healthy control ECO, whereas this protein was absent in CF-ECO (**A**). Instead, only an immature CFTR (band of 130 kDa) was present in CF-ECO.The immature band was also detected in healthy ECO organoids and in a T84 control cell line (colon carcinoma cell line expressing mature CFTR). Shown is a cropped blot and b-actin served as a loading control. The full blot is provided in the supplemental information file. The CF-ECO showed similar cell proliferate speed as healthy ECO; 18% at passage 15 as demonstrated with EdU incorporation using flow cytometry (**B**). Functional transporter assays were done using CF-ECO (blue line) and healthy ECO (pink line) in an Ussing chamber assay (**C**). As expected, CF-ECO cells do not respond to CFTR stimulation with forskolin, whereas healthy ECO stimulated with forskolin showed a clear current which was inhibited by CFTR inhibitors, GlyH-101 and pyrimido-pyrrolo-quinoxalinedione (PPQ). These inhibitors had no effect on the CF-ECO. Stimulation with UTP shows functionality of other Ca^2+^-dependent Cl^-^ channels in both ECO (CF and healthy) and both are responsive to the specific inhibitor of this channel, T16A-inh-A01. Representative cultures of undifferentiated CF-ECO (upper panel, **D**) and CF-ECO that are differentiated towards cholangiocytes (DM-chol, lower panel, **D**). Changes in gene expression of CF-ECO after cholangiocyte-fate differention (**E**). Changes are similar to healthy ECO. Functional transport of Rhodamine123 via transported channels was shown in CF-ECO organoids differentiated towards cholangiocyte fate (**F**). This signal is specific for MDR1 as it could be complete blocked by Verapamil. Shown is one of two experiments and MDR1 activity was comparable to healthy ECO.

To determine the feasibility to use ECO as a model for bile duct diseases, organoids were initiated from eBD tissue collected from a CF patient at time of liver transplantation. This patient was known to carry a compound heterozygous mutation in the CFTR gene, resulting in a non-functional CFTR protein. Western blot analysis of protein lysate from organoids confirmed the absence of mature CFTR protein (band at 170 kDa) in the CF-ECO (Fig. 6A) which was clearly visible in the healthy ECO. Immature CFTR (band at 130 kDa) was detected in both CF and healthy organoids as expected. Initiation and expansion of the CF-ECO was similar efficient compared to ECO from healthy donors. As shown in Fig. 6B, EdU incorporation assays showed similar levels level of cell proliferation, with 18% of cells S-phase for CV-ECO, as compared to healthy ECO (14%, Fig. 1J). Functional transporter assays in Ussing chambers showed a clear lack of forskolin-activated CFTR-mediated chloride currents (Fig. 6C). To demonstrate that this non-responsiveness was specific for the CFTR channel, the Ca^2+^ activated Cl^-^ channel was activated with UTP at the apical side of the cells. Upon UTP addition, the channel was clearly activated in the CF-ECO cells and could be specifically inhibited by CaCC channel inhibitor T16inh-A01. As previously showed in Fig. 3, ECO of healthy donors show clear activity of both CFTR and the Ca^2+^ activated Cl^-^ channel.

The CFTR channel is known to co-localized with other transporter channels, like MDR1^23^. To test whether the CFTR deficiency in organoids effects the function of other channels we needed to differentiate CF-ECO in DM-chol condition in order to measure MDR1 activity. As shown in Fig. 6D, the CF-ECO showed comparable cholangiocyte-fate differentiation as the healthy counterparts. The morphological changes in CF-ECO appeared smaller. In DM-chol conditions less dense organoids were observed as compared to healthy donor ECO (Fig. 4). This difference could be attributed to changes in water export due to dysfunctional CFTR channel activity. As shown in Fig. 6E, changes in gene expression of CF-ECO in DM-chol condition were similar to normal ECO (Fig. 4). Stem cell and proliferation markers, LGR5 and Ki67 were down regulated upon differentiation. Conversely, expression of mature cholangiocyte markers KRT19, NOTCH2, and HNF1B were upregulated (Fig. 6E). Although the MDR1 pump and the CFTR channel are co-localized^23^, MDR1 functionality was not diminished in ECO of CF patient (Fig. 6F). Efficient transport of the Rhodamine123 was seen in the CF-ECO which again could be blocked by specific inhibitor, Verapamil. Combined these results provide evidence that ECO can be used as a patient-specific model for bile duct diseases like CF and encourage further research on CF physiology and personalized drug testing.

## Discussion

The development, homeostasis and repair of iBD and eBD involves distinct mechanisms, either involving cholangiocyte or progenitor cell proliferation or maturation. In this study, organoids were successfully initiated from human eBD and pair-wise compared to the well-established iBD-organoids^14^. Overall these two types of organoids, ECO and ICO, behaved highly similar in terms of gene expression, proliferation capacity and function capacities. Despite these similarities, we observed regional-specific differences in their differentiation potential. As reported earlier, ICO have clear bipotent differentiation capacity. However, here we found that ECO are committed to differentiation towards cholangiocyte-fate and lack the ability to differentiate towards a hepatocyte-like phenotype. A similar observation was recently reported by Rimland et al*.*^17^. They also demonstrate that differences exist between ICO and ECO which is mostly reflected in the exclusive capacity of ICO to differentiate in the direction of hepatocyte lineage. Considering the differences in embryonic origin ECO may also be bipotent, not in respect to hepatocyte-fate, but with the potential for pancreatic-lineage and cholangiocyte-lineage differentiation potential. Although this has also been suggested by others^6,7^, it requires further research to confirm this. As shown in Fig. 2, only few genes were differentially expressed between ECO and ICO lines. Currently, a follow-up study has started to find markers that distinguish between the organoid-initiating cells of each type at a single-cell resolution and show their localization and distribution in primary liver and bile duct tissue.

Recent advances in stem cell biology and novel culture technology enabled the large-scale expansion of primary biliary epithelium by generating complex 3D stem cell-derived constructs or organoids from liver biopsies^14^. Whether these organoids arise from LGR5-positive or negative cells, remains to be determined. Lineage tracking studies in Lgr5-creERT2 reporter mice suggests that LGR5-negative cells could be activated upon liver injury induction and at that point start to express LGR5^24^. This implies plasticity of liver ductal cells that respond to the need of parenchymal cells.

These liver-organoids, or more specifically, ICO enabled detailed analysis of intrahepatic biliary epithelium. In parallel, mature cholangiocytes were derived from human induced pluripotent cells (iPSC) to act as model for biliary disease and drug screening^25^. However, both liver-derived organoids and iPSC-based cholangiocytes could not provide insight into the (stem) cell populations present in eBD. With the availability of eBD biopsies collected during liver transplantation, we embarked on characterizing organoids from the extrahepatic biliary epithelium (eBD) and compared them to paired organoids initiated from iBD of the same donor. The non-canonical Wnt-stimulated extrahepatic cholangiocyte organoids as described by Sampaziotis^16^, isolated from eBD are potent cells for regenerative medicine applications^26,27^. However, these cholangiocyte organoids are assumedly derived from mature primary cholangiocytes (not stem/progenitor cells) and are not dependent on canonical Wnt signaling like the LGR5-positive ICO^14^ and ECO. Although cholangiocyte organoid cultures are interesting in their own right they are not representative of extrahepatic LGR5-positive stem cell populations which reside in the peribiliary glands and which are dependent on canonical Wnt signaling^28,29^. As the ECO resemble biliary progenitor cells that are present in the bile ducts, they can specifically be employed in studies focusing on stem cell defects leading to biliary diseases and developmental biology research.

The results described in this study may reflect intrinsic differences between intra- and extrahepatic resident (stem/progenitor) cells. It is known that differences in phenotype and functionality between mature cholangiocyte populations depend on their localization along the biliary tree^17,19^. With the development of novel technology as single cell RNA sequencing, now the cellular composition of the liver can be reconstructed based on distinct gene expression profiles of individual cell types^15,30^. Sequencing single EpCAM-positive liver cells showed they are a heterogeneous set of cells including mature cholangiocytes as well as potential progenitor cell subpopulations which both express cell surface markers TROP2 (or TACSTD2), FGFR2, TM4SF4, and CLDN1. Further analyses indicated that the cell population with intermediate expression of TROP2 (EpCAM^+^ TROP2^int^) to indicate the true progenitor population with both hepatocyte and cholangiocyte differentiation potential^15^. Interestingly, an earlier study on TROP2 in eBD reported that though TROP2 is non expressed in peribiliary glands, upon damage of the bile ducts TROP2 is clearly upregulated in these glands epithelial cells^31^. Although novel culture techniques provide evidence for long-term expansion of primary hepatocytes^32,33^ and primary cholangiocytes^26,27^, both cell types remain challenging to maintain in culture while keeping their mature functions^13,14,28^. Expansion of ECO could provide in this shortcoming. In addition to cell expansion for use in tissue engineering, as was recently demonstrated by recellularization of decellularized human eBD^34^, we demonstrate that ECO, have the potential to be used as a model for bile duct-related diseases as CF. To show proof of concept, ECO were cultured from eBD tissue from a CF patient and assessed for disease-typical features. Transporter channels were functional in ECO derived from healthy donor bile duct as demonstrated by activating and blocking specific transporter function (CFTR and MDR1^23,35^), whereas CFTR channel activity was impaired in the CF patient-derived ECO. Of note, we also showed that healthy ECO respond significantly higher to forskolin-activation as ICO, indicating that ECO better mimic biliary epithelium that is affected in CF patients. Modeling diseases as CF using ECO will help to study not only pathways involved in fluid transport and epithelial function, but also provide the opportunity to test (toxic) effects of bile acids and beneficial effects of CFTR targeted drugs.

In conclusion, this study shows the feasibility to culture Wnt-driven organoids from small tissue biopsies of human eBD and that these ECO can be differentiated towards functional cholangiocyte-like cells. This opens the avenue for use in pre-clinical toxicology studies, development and testing of novel treatments in a personalized setting, and tissue engineering for patient-specific clinical applications.

## Material and methods

### Human organoid culture initiation and expansion

Tissue samples (≤ 0.5 cm^3^) of donor (extrahepatic) common bile duct (eBD) and donor liver biopsies (n > 40) were collected during liver transplantation at the Erasmus Medical Center Rotterdam. Use of both tissues for research purposes was approved by the Medical Ethical Council of the Erasmus MC and informed consent was given (MEC-2014-060). Liver and eBD biopsies were obtained from a 17-year old male Cystic Fibrosis (CF) patient undergoing liver transplantation at the University Medical Center Groningen. Informed consent was obtained to use these biopsies for research (STEM Study—protocol number 1-402/K, informed consent was obtained from a parent). This patient was compound heterozygous for F508del and R1162X, resulting in a non-functional CFTR transporter. Biopsies were stored in organ preservation fluid (University of Wisconsin Belzer UW Cold Storage Solution, Bridge of Life Ltd. London, UK) and transported at 4 °C. The biopsies were used to initiate ECO and ICO cultures which were initiated as described for human liver tissue^14,36^. In short, the biopsies (liver and eBD) were washed in Advanced DMEM/F-12 medium supplemented with penicillin/streptomycin (with 10,000 U/ml, Life Technologies), HEPES (1 M, Life Technologies), Ultraglutamine (200 mM, Life Technologies). Both liver and eBD biopsies were minced and digested in a collagenase solution (2.5 mg/ml collagenase Type A, Sigma Aldrich in Advanced DMEM/F-12 medium), rocking at 37 °C for 20 min. Single cell solutions were gained by passaging over a 70 μm nylon mesh cell strainer and washed in Advanced DMEM/F-12 medium (5 min, 1500 rpm at 4 °C) after which the pellet was diluted in ice cold Matrigel (Corning) and seeded in 25 μl droplets in 48-wells plates. After solidifying of the Matrigel, 250 μl culture initiating medium was added and cells were incubated at 37˚C, 5% CO2 for three days. Culture initiating medium consisted of Advanced DMEM/F12 medium (Invitrogen) supplemented with 1% N2, 1% B27 (both Gibco), 1.25 mM N-Acetylcystein (Sigma), 10 nM gastrin (Sigma), 50 ng/ml EGF (Peprotech), 100 ng/ml FGF10 Peprotech), 25 ng/ml HGF (Peprotech), 10% R-spondin (conditioned medium), 10 nM nicotinamide (Sigma), 5 µM A83.01 (Tocris), 10 µM forskolin (Tocris), 25 ng/ml Noggin (conditioned medium), 30% Wnt (conditioned medium), 10 µM Y27632 (Sigma), and hES cell cloning recovery solution (Stemgent). After 3 days, the initiation medium was changed to expansion medium (EM), deprived of noggin, Wnt, Y27632 and hES cell cloning recovery solution. Organoid formation was seen within 2 or 3 days after culture initiation and medium was refreshed every 3 days. Organoids were split (1:3) every 7 days. To prepare viably frozen stocks (from different passages), organoids were mechanically dissociated and mixed with recovery cell culture freezing medium (Gibco) according to the manufacturers protocol and stored at − 196 °C.

### DNA-synthesis and cell cycle analysis

Cell proliferation was analyzed by direct measurement of DNA synthesis using the Click-iT EdU Alexa Fluor 488 Flow Cytometry kit (Thermo Fisher). For this, organoids of different passage numbers were incubated with the thymidine analogue 5-ethynyl-2′-deoxyuridine (EdU) during 4 h at 37 °C, 5% CO_2_ after which the organoids were harvested and made single cell using trypsin–EDTA incubation (15 min at 37 °C). The cells were further processed according to the manufacturer’s protocol. The percentage of S-phase cells in the organoids was determined using standard flow cytometry methods (FACSCalibur, BD Biosciences).

### Organoid growth rate analysis

Organoid growth rates were determined using a custom-made pipeline for bright field-based image segmentation (Hof et al., *in submission*) in three pairs of ICO and ECO (passage 7–16). In brief, organoids were split, seeded in 5 µl of Matrigel into 96-well plates at similar densities, overlaid with 100 µl of expansion medium, and were cultured for 84 h before imaging. Organoids were imaged for 30 h at 1 h intervals using bright field microscopy (microscope: Zeiss AxioObserver.Z1; objective lens: Zeiss Plan-Apochromat 5×/0.16; tiling: 2 × 2; z-planes: 10; z-spacing: 65 µm). The recorded time-lapse image stacks were pre-processed with Fiji [ImageJ version 1.51n, Java version 1.8.0_6 (64-bit)] by reducing the dimensionality of the raw data set from 4 (2 × 2) tiles with 10 z-planes each to 1 stitched image with 1 z-plane per time frame using the plugin Grid/Collection stitching^37^. The resulting image stacks were subsequently segmented based on projected luminal areas of the organoids by using the Fiji plugin Morphological Segmentation (MorphoLibJ)^38^. Segmented luminal areas were measured with the Fiji plugin Region Morphometry (MorphoLibJ)^38^. The luminal areas of individual organoids were normalized to the average luminal area over the first 5 time-points. Organoids which were not detected in all 31 time frames were manually excluded from the analysis. Data is displayed as mean ± SEM in the corresponding graph.

### Hepatocyte differentiation

Organoids were expanded for 5–7 days before initiation of hepatocyte differentiation. Differentiation towards hepatocyte fate was initiated with the addition of 25 ng/ml BMP7 (Peprotech) to the expansion medium and lasted 5 days. Subsequently, organoids were passaged with a 1:1.5 split ratio and medium was changed to human hepatocyte differentiation medium based on the original differentiation protocol as published by Huch et al*.*^14^ and Gehart et al*.* (World Interlectual Property Organization Patent WO2017149025A1). Medium was changed every 2–3 days for a total of 10 days after which the organoids were analyzed for hepatocyte-specific markers.

### Cholangiocyte differentiation

The protocol used to differentiate organoids towards cholangiocytes was adapted from the previously published protocol for iPSC differentiation towards cholangiocytes^25^. From the last steps onwards, describing the differentiation from the hepatoblast phenotype to cholangiocytes, this protocol was used to differentiate the eBD and iBD organoids towards cholangiocytes. In detail, organoids were expanded for 7 days in Matrigel to near-full wells. Culture medium was then switched to differentiation medium, consisting of AdDMEM/F12 supplemented with B27 (1×, Gibco), fibroblast growth factor 10 (FGF10, 50 ng/ml, Peprotech), Activin-A (50 ng/ml, Gibco), and retinoic acid (3 μM, Sigma-Aldrich). After 4 days, medium was changed to William’s E medium (Gibco, Life Technologies) supplemented with nicotinamide (10 mM, Sigma-Aldrich), sodium bicarbonate (17 mM, Sigma-Aldrich), 2-phospho-i-ascorbic acid tri-sodium salt (0.2 mM, Sigma-Aldrich), sodium pyruvate (6.3 mM, Invitrogen), glucose (14 mM, Sigma-Aldrich), HEPES (20 mM, Invitrogen), ITS + premix (BD Biosciences), dexamethasone (0.1 M, R&D Systems), epidermal growth factor (EGF, 20 ng/ml, R&D Systems), Ultraglutamine (2 mM, Invitrogen), and penicillin (100 U/ml)/ streptomycin (100 g/ml). Differentiation was blocked by adding 50 μM DAPT (Sigma) to the culture medium. The medium was refreshed every 2 days for a total of 10 days after which the organoids were analyzed.

### RNA sequencing

Organoids (ICO and ECO, before and after differentiation towards the hepatocyte and cholangiocyte phenotypes) were collected in lysis buffer (QIAzol lysing reagent, Qiagen) and total RNA was collected using the miRNeasy mini kit (Qiagen). The amount and integrity of RNA was analyzed using the Agilent 2100 Bioanalyser (Agilent). RNA sequencing libraries were generated from 100 ng of total RNA using the TruSeq Stranded Total RNA Library Prep Human/Mouse/Rat kit (Illumina) and sequenced 2 × 75 bp on the Illumina Nextseq500. Sequencing reads were subsequently aligned against the human reference genome (GRCh37) using STAR 2.4.2a 21and counted with HTSeq 0.6.1 22. Differential expression analysis was done using DESeq 1.20.0 23. Genes were considered to be significantly differentially expressed when *p* adjusted < 0.05 (Benjamini Hochberg FDR correction). RNA sequencing data was submitted to EGA under study ID: EGAS00001003792. To access: Username: reviewer22677@ebi.ac.uk, password: unYGgKju. The GSEA analysis was performed using the software from Broad Institute (v4.0.3).

### Forskolin-indiced swelling assay (FIS)

To assess CFTR functionality, Forskolin-Induced Swelling (FIS) of organoids was performed essentially as described by Dekkers et al.^39,40^. In brief, ICO and ECO were seeded in 96-well cell culture plates (circa 50/well), in a 5 µL droplet of Matrigel. After 2 days, organoids were loaded with calcein-green (Invitrogen; acetoxymethyl ester; 5 µmol/l; 1 h) suspended in modified Meyler solution. Calcein-fluorescent, viable organoids were maintained at 37 °C in 5% CO_2_, and visualized on a confocal microscope equipped with a computer-controlled moveable stage (5× objective; Leica TCS SP5). After CFTR phosphorylation/activation was triggered by addition of forskolin (5 µM), images were acquired at 6 min intervals for 2 h. CFTR-mediated anion secretion stimulates osmotic fluid transport into the enclosed organoid lumen, leading to a volume increase. To assess organoid swelling, the area enclosed by the fluorescent cells lining the organoids was quantified on the ImageJ platform (NIH, USA), using a software module developed by the Optical Imaging Center of the Erasmus MC, which fully automates image analysis. Data depict the cumulative increase in volume over 1 h. In the graphic display of the data, each data point represents the average of triplicate wells.

### Ussing chamber assay

Ussing chamber assays were performed essentially as described elsewhere^41^. In short, organoids were dissociated into single cells and seeded on a permeable support (Transwell 3470; Corning) that were coated with Matrigel (1:20 in PBS), and cultured in EM organoid medium. After the cells had formed a confluent monolayer (circa 10 days after seeding), filters were inserted in Ussing chambers (P2302T/P2300; Physiologic Instruments, San Diego, CA), and bathed in modified Meyler solution (128 mM NaCl, 4.7 mM KCl, 1.3 mM CaCl_2_, 1.0 mM MgCl_2_, 0.3 mM Na_2_HPO_4_, 0.4 mM NaH_2_PO_4_, 20 mmol/l NaHCO_3_, 10 mM HEPES), supplemented with glucose (10 mM), in 95% O_2_, 5% CO_2_, pH 7.3, at 37 °C. The transepithelial potential difference (PD) was clamped at 0 mV using a VCC MC8 voltage clamp module (Physiologic Instruments), and the resulting short-circuit current (Isc) was digitally recorded using an analog-to-digital signal converter and associated software (Acquire and Analyze 2.3; Physiologic Instruments). Anion secretion was stimulated by addition of the adenylyl cyclase activator forskolin (10 µmol/l, Sigma-Aldrich) or the purinergic receptor agonist UTP (50 µmol/l, Sigma-Aldrich) to the luminal bathing solution. Forskolin-induced and UTP-induced secretion was inhibited by Glyh-101 (20 µmol/l, Sigma-Aldrich) and T16inh-A01 (50 µmol/l, Sigma-Aldrich), respectively.

### Rhodamine 123 assay

To determine the presence of calcium channels, including multidrug resistance protein 1B (MDR1b—important in the biliary excretion of large hydrophobic components—member of the P-glycoprotein membrane transporter family), Rhodamine 123 (Rh123) was added to the cultures. Rh123 is a fluorescent chemical compound that can be transported by these transporter channels 27. For this, cold Advanced DMEM/F12 medium was used to remove Matrigel and collagen from the cultures that were subsequently pretreated with DMSO or 10 μM Verapamil (Sigma-Aldrich—MDR1b inhibitor) for 30 min, followed by 5 min of incubation with 100 μM Rh123 (Sigma-Aldrich). The organoids were washed 3 times with expansion medium. Fluorescence (excitation wavelength: 511 nm; emission wavelength: 534 nm) was visualized with a Leica SPE-II confocal system, 30 min after washing and analyzed with ImageJ.

### Flow cytometry

To assess TROP2 expression, paired ICO and ECO (passage 5) from three donors were dissociated using Trypsin/EDTA (15 min at 37 °C), washed in 8 ml in Advanced DMEM/F-12 medium (1500 rpm, 5 min, 4 °C) and cells were re-suspended in EBSS to make a single cell suspension. TROP2 antibody (Invitrogen; rabbit monoclonal conjugated to Alexa Fluor-488, clone MR54, used 1:100) was added (30 min, on ice) and cells were subsequently measured on a FACS Canto flow cytometer (BD Biosciences).

Flow cytometric analysis of the starting cell populations, liver (n = 3) and extrahepatic bile duct biopsies (n = 3), collected during liver transplantation were treated similar to organoid initiation as described above. Instead of addingMatrigel to initiate the organoid cultures, the cells were stained for flow cytometry using monoclonal antibodies against human LGR5 (1:100, Clone 8F2, BD Biosciences) and human EpCAM (1:100, Clone 9C4. Biolegend), conjugated directly with PerCP and PerCP/Cy5.5, respectively. Cells were measured on a FACS CantoII flow cytometer (BD Biosciences) and analysed using FACS Diva software. Cells from ICO and ECO were harvested by trypsin-treatment (10 min, 37 °C), subsequently washed in Advanded DMEM/F-12 and stained with EpCAM and LGR5 to be analysed by flow cytometry.

### Immunohistochemistry

Organoids were formalin-fixed (4%) for 24 h, paraffin-embedded and sectioned (5 µm) for histological examination. To analyze the general morphology, sections were stained with hematoxylin–eosin (H&E) and analyzed using a bright field microscope. Additional sections were processed and stained using standard IHC protocols with specific antibodies for cytokeratin 19 (KRT19) and Mucin-1 (Muc-1). After deparaffinization, antigen retrieval was performed in a citrate buffer (pH 6) at boiling temperatures for 10 min. Subsequently, sections were incubated with primary antibodies (Muc-1 clone E29, Fisher Scientific 1:500, and KRT-19 clone RCK108, Agilent Dako, 1:250) overnight at 4 °C. After washing, sections were incubated with secondary antibody (Envision Flex HRP, Agilent) for 60 min at RT prior to incubation with DAB substrate. Slides were analyzed with a bright field microscope and imaged with a Nikon DS camera. To determine cell proliferation, stainings were done on sections using standard immunofluorescence (IF) protocols, using a specific antibody for Ki67 (1:100, kind gift from PARTS, Department of Pathology, Erasmus MC, Rotterdam), *CFTR Western blot analysis.*

To demonstrate the near absence of the CFTR protein in the CF-eBD organoids, Western blot analysis was performed. Cells from ECO derived from CF patients and healthy donors were harvested, collected by centrifugation, and lysed in ice-cold RIPA buffer (Cell Signaling Technology) supplemented with a protease inhibitor cocktail (Roche). Total protein content was measured photometrically using the RC-DC protein assay (Bio-Rad Laboratories, Inc., CA). Cell lysates were subjected to sodium dodecylsulfate polyacrylamide gel electrophoresis (SDS-PAGE) (8.0% acryl amide), and proteins were transferred to polyvinylidene fluoride membrane (120 V, 70 min). Nonspecific binding sites were blocked for with Western blot blocking buffer (1 h, RT; ThermoFisher Scientific). The CFTR primary antibodies ‘570’ and ‘596’ antibodies were obtained through the Cystic Fibrosis Foundation Therapeutics initiative (www.cftrfolding.org/CFFTReagents.htm) and used at a 1:400 dilution in Western blot blocking buffer (16 h, at 4 °C)^39,42^. After washing in Tris-buffered saline with Tween-20 (TBST), the membrane was incubated with donkey polyclonal anti-mouse immunoglobulin (Ig)G-H&L HRP (Abcam, 1:10.000; 1 h) in Western blot blocking buffer. After washing, the reaction was visualized using Chemiluminescence (ChemiDoc, Bio-Rad). Normalization was done to ß-actin (C4 HRP, sc-47778, Santa Cruz Biotechnology, USA) expression using Image Studio lite version 5.2 (LI-COR Biosciences, NE).

### Statistical analysis and data access

All values are represented as mean ± SEM. All statistical analysis were performed using GraphPad Prism 7, R statistical environment or Python 3 with NumPy, Pandas and SciPy libraries 28. A two-sided Student’s t-test was done to test statistical significance between means (qRT-PCR) and Two-tailed t-test was performed to analyse relative ICO and ECO sizes. A *p* value < 0.05 was considered statistically significant. RNA sequencing data was submitted to EGA under study ID: EGAS00001003792.

All methods were carried out in accordance with relevant guidelines and regulations of the Erasmus MC—University Medical Center Rotterdam and The Netherlands.

## Supplementary information

Supplementary Information.

## References

[1] Boulter L (2012). Macrophage derived Wnt signalling opposes Notch signalling in a Numb mediated manner to specify HPC fate in chronic liver disease in human and mouse. Nat. Med..

[2] Lu W-Y (2015). Hepatic progenitor cells of biliary origin with liver repopulation capacity. Nat. Cell Biol..

[3] Boulter L, Lu W-Y, Forbes SJ (2013). Differentiation of progenitors in the liver: a matter of local choice. J. Clin. Investig..

[4] Tabibian JH, Masyuk AI, Masyuk TV, O'Hara SP, LaRusso NF (2013). Physiology of cholangiocytes. Compr. Physiol..

[5] de Jong IEM, van Leeuwen OB, Lisman T, Gouw ASH, Porte RJ (2018). Repopulating the biliary tree from the peribiliary glands. Biochim. Biophys. Acta Mol. Basis Dis..

[6] Si-Tayeb K, Lemaigre FP, Duncan SA (2010). Organogenesis and development of the liver. Dev. Cell.

[7] Raynaud P, Carpentier R, Antoniou A, Lemaigre FP (2011). Biliary differentiation and bile duct morphogenesis in development and disease. Int. J. Biochem. Cell Biol..

[8] Maroni L (2015). Functional and structural features of cholangiocytes in health and disease. Cell Mol. Gastroenterol. Hepatol..

[9] Benedetti A, Bassotti C, Rapino K, Marucci L, Jezequel AM (1996). A morphometric study of the epithelium lining the rat intrahepatic biliary tree. J. Hepatol..

[10] Alpini G (1996). Morphological, molecular, and functional heterogeneity of cholangiocytes from normal rat liver. Gastroenterology.

[11] Sato K, Meng F, Giang T, Glaser S, Alpini G (2018). Mechanisms of cholangiocyte responses to injury. Biochim. Biophys. Acta BBA Mol. Basis Dis..

[12] Clevers H, Loh KM, Nusse R (2014). Stem cell signaling. An integral program for tissue renewal and regeneration: Wnt signaling and stem cell control. Science.

[13] Huch M (2013). In vitro expansion of single Lgr5+ liver stem cells induced by Wnt-driven regeneration. Nature.

[14] Huch M (2015). Long-term culture of genome-stable bipotent stem cells from adult human liver. Cell.

[15] Aizarani N (2019). A human liver cell atlas reveals heterogeneity and epithelial progenitors. Nature.

[16] Sampaziotis F (2017). Reconstruction of the mouse extrahepatic biliary tree using primary human extrahepatic cholangiocyte organoids. Nat. Med..

[17] Rimland, C.A.*, et al.* Regional differences in human biliary tissues and corresponding in vitro derived organoids. *Hepatology*, online ahead of print (2020). 10.1002/hep.31252 PMC8641381 32222998

[18] Schmelzer E (2007). Human hepatic stem cells from fetal and postnatal donors. J. Exp. Med..

[19] Raven A (2017). Cholangiocytes act as facultative liver stem cells during impaired hepatocyte regeneration. Nature.

[20] Ng-Blichfeldt J-P (2018). Retinoic acid signaling balances adult distal lung epithelial progenitor cell growth and differentiation. EBioMedicine.

[21] Barman PP, Choisy SCM, Gadeberg HC, Hancox JC, James AF (2011). Cardiac ion channel current modulation by the CFTR inhibitor GlyH-101. Biochem. Biophys. Res. Commun..

[22] Schneeberger K (2020). Large-scale production of LGR5-positive bipotential human liver stem cells. Hepatology.

[23] Scoazec JY (1997). The plasma membrane polarity of human biliary epithelial cells: in situ immunohistochemical analysis and functional implications. J. Hepatol..

[24] Cao W (2017). Dynamics of proliferative and quiescent stem cells in liver homeostasis and injury. Gastroenterology.

[25] Sampaziotis F (2015). Cholangiocytes derived from human induced pluripotent stem cells for disease modeling and drug validation. Nat Biotechnol.

[26] Sampaziotis F (2018). Building better bile ducts. Science.

[27] Justin AW, Saeb-Parsy K, Markaki AE, Vallier L, Sampaziotis F (2018). Advances in the generation of bioengineered bile ducts. Biochim. Biophys. Acta (BBA) Mol. Basis Dis..

[28] Huch, M.*, et al. Unlimited In Vitro Expansion of Adult Bi‐potent Pancreas Progenitors Through the Lgr5/R‐spondin axis* (2013). 10.1038/emboj.2013.204 PMC3801438 24045232

[29] Huch M, Boj S, Clevers H (2013). Lgr5+ liver stem cells, hepatic organoids and regenerative medicine. Regen. Med..

[30] MacParland SA (2018). Single cell RNA sequencing of human liver reveals distinct intrahepatic macrophage populations. Nat. Commun..

[31] Matsui S (2018). Characterization of peribiliary gland-constituting cells based on differential expression of trophoblast cell surface protein 2 in biliary tract. Am. J. Pathol..

[32] Hu H (2018). Long-term expansion of functional mouse and human hepatocytes as 3D organoids. Cell.

[33] Zhang K (2018). In vitro expansion of primary human hepatocytes with efficient liver repopulation capacity. Cell Stem Cell.

[34] Willemse J (2020). Scaffolds obtained from decellularized human extrahepatic bile ducts support organoids to establish functional biliary tissue in a dish. Biotechnol. Bioeng..

[35] Hoof T, Demmer A, Hadam MR, Riordan JR, Tummler B (1994). Cystic fibrosis-type mutational analysis in the ATP-binding cassette transporter signature of human P-glycoprotein MDR1. J. Biol. Chem..

[36] Broutier L (2016). Culture and establishment of self-renewing human and mouse adult liver and pancreas 3D organoids and their genetic manipulation. Nat. Protoc..

[37] Preibisch S, Saalfeld S, Tomancak P (2009). Globally optimal stitching of tiled 3D microscopic image acquisitions. Bioinformatics.

[38] Legland D, Arganda-Carreras I, Andrey P (2016). MorphoLibJ: integrated library and plugins for mathematical morphology with ImageJ. Bioinformatics.

[39] Dekkers JF (2013). A functional CFTR assay using primary cystic fibrosis intestinal organoids. Nat. Med..

[40] Vidovic D (2016). rAAV-CFTRDeltaR rescues the cystic fibrosis phenotype in human intestinal organoids and cystic fibrosis mice. Am. J. Respir. Crit. Care Med..

[41] Bijvelds MJ, Bot AG, Escher JC, De Jonge HR (2009). Activation of intestinal Cl-secretion by lubiprostone requires the cystic fibrosis transmembrane conductance regulator. Gastroenterology.

[42] van Meegen MA (2013). CFTR-mutation specific applications of CFTR-directed monoclonal antibodies. J. Cyst. Fibros..

