# Supplementary material for: Human extrahepatic and intrahepatic cholangiocyte organoids show region-specific differentiation potential and model cystic fibrosis-related bile duct disease
Source: Sci Rep. 2020 Dec 14;10:21900. doi: 10.1038/s41598-020-79082-8 (PMC7736890; doi:10.1038/s41598-020-79082-8)

**Supplemental information file to:**

Human extrahepatic and intrahepatic cholangiocyte organoids show region-specific differentiation potential and model cystic fibrosis-related bile duct disease

Monique M.A. Verstegen^1*^, Floris J.M. Roos^1**^, Ksenia Burka^1**^, Helmuth Gehart^2^, Myrthe Jager^3^, Maaike de Wolf^1^, Marcel J.C. Bijvelds^4^, Hugo R. de Jonge^4^, Arif I. Ardisasmita^6^, Nick A. van Huizen^1,7^, Henk P. Roest^1^, Jeroen de Jonge^1^, Michael Koch^5^, Francesco Pampaloni^5^, Sabine A. Fuchs^6^, Imre F. Schene^6^, Theo M. Luider^7^, Hubert P.J. van der Doef^8^, Frank A.J.A. Bodewes^9^, Ruben H.J. de Kleine^9^, Bart Spee^10^, Gert-Jan Kremers^11^, Hans Clevers^2^, Jan N.M. IJzermans^1^, Edwin Cuppen^3^, Luc J.W. van der Laan^1^

Included are:
- Supplemental Figure S1
- Supplemental Figure S2
- Supplemental information


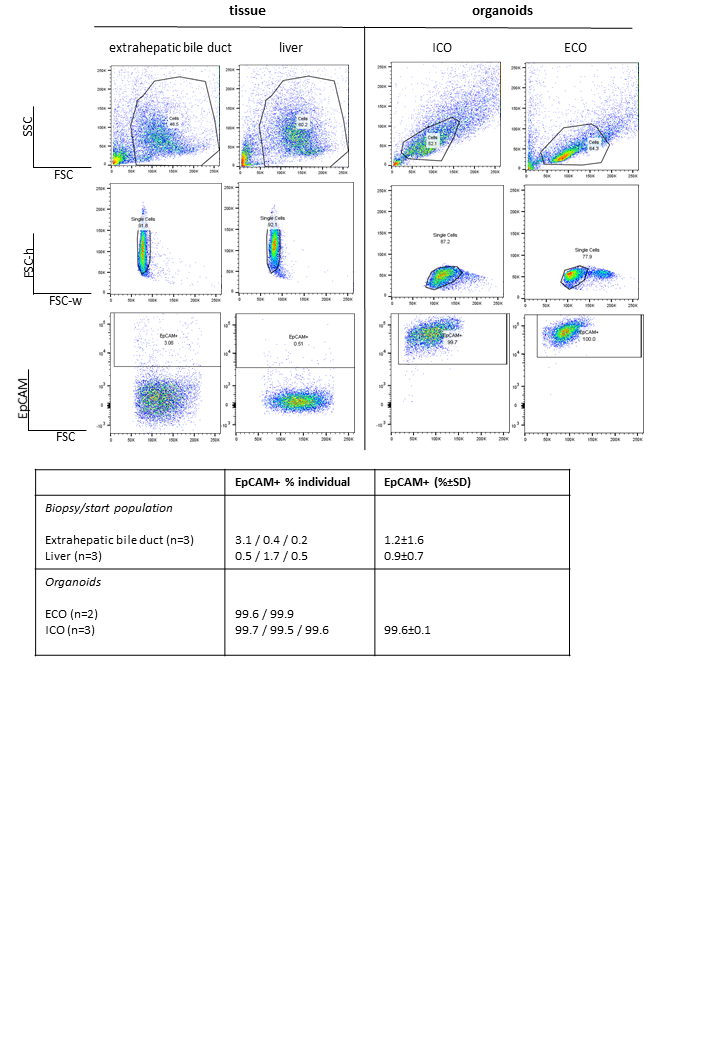


*Supplemental Figure S1. Representative dotplots of flow cytometric analysis. Biopsies of donor liver (n=3) and extrahepatic bile duct (n=3), as well as ICO (n=3) and ECO (n=2) were assessed for the expression of EpCAM positive cells by flow cytometry. Gating strategy is shown by the scatter pattern gate, and single cells gating (FCS-h vs FSC-w). Average EpCAM positivity (percentage of life single-cell events ± SD) is presented in the table.*

**
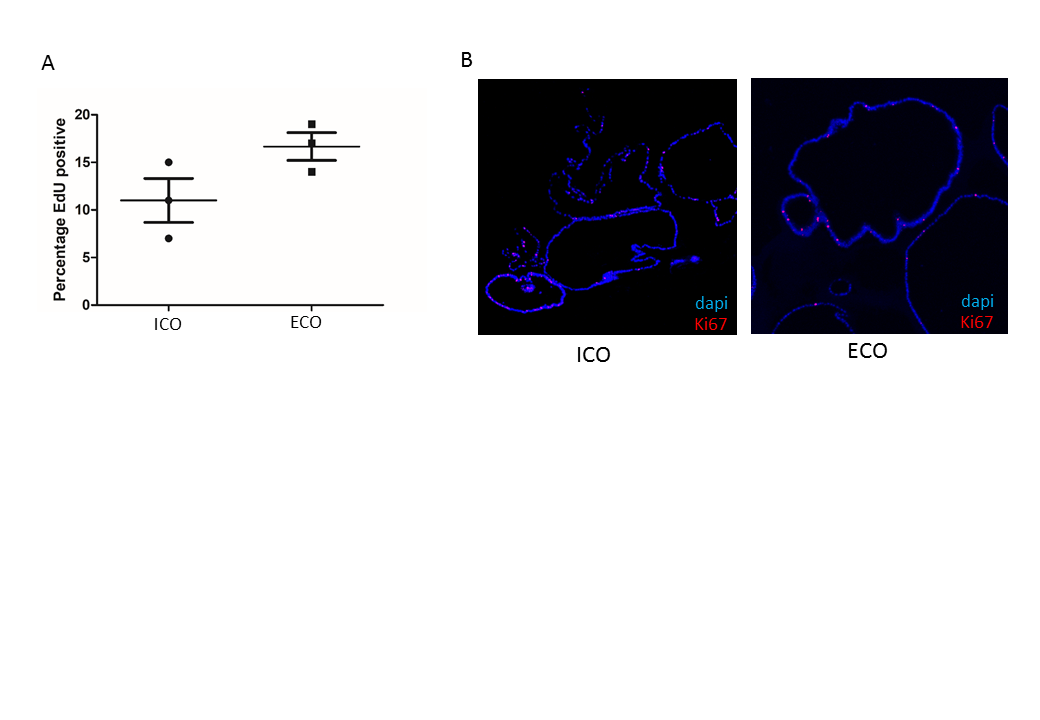
**

*Supplemental Figure S2. Proliferation rate of ECO and ICO. (A) EdU incorporation was analysed in paired ECO and ICO from the same donor (n=3) by flow cytometry. The average of proliferating cells (S-phase) was 11.0±2.1% and 17.3±2.4% (mean±SEM) in ICO and ECO, respectively. Differences were not significantly different between these organoid types (p= 0.122). To confirm, FFPE sections of ECO and ICO were stained with a monoclonal antibody for the proliferation marker Ki67 using immunofluorescence (B). Representative images show a similar Ki67positive cells in both organoid types (red = Ki67, blue = dapi).*

Supplemental information

Figure 6A shows a Western blot analysis. We show the cropped details of the blot in the manuscript. In this supplemental file, we provide the complete Western blot, according to standard procedures for Scientific Reports.


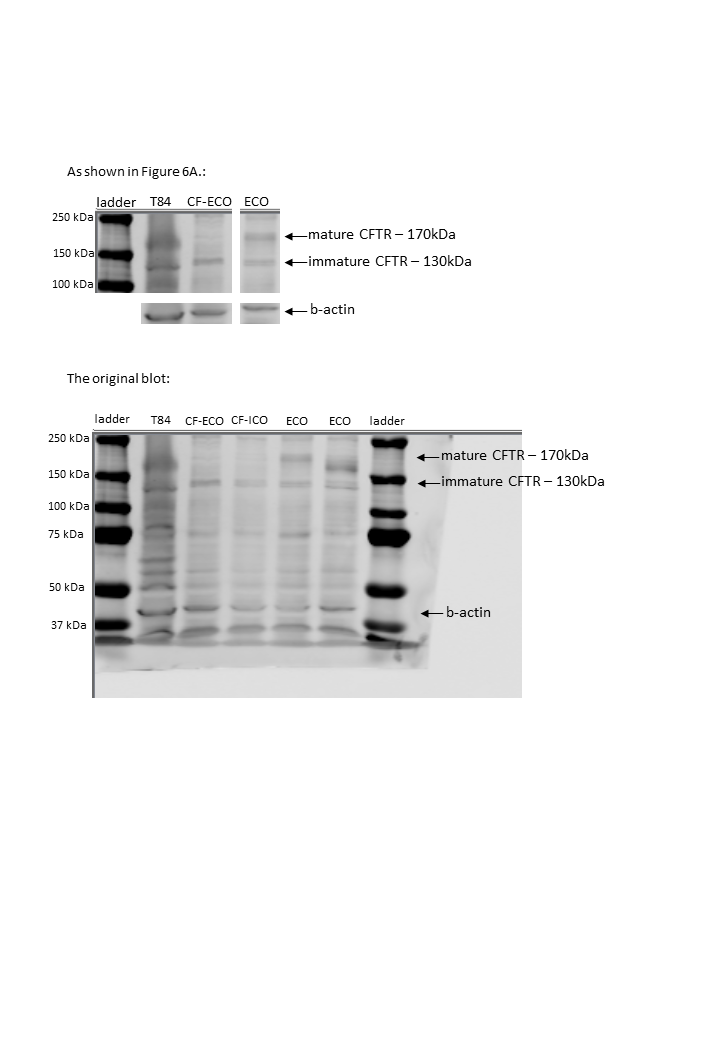

Supplement: Supplementary file 1 — Supplementary Information. [file 41598_2020_79082_MOESM1_ESM.docx]
